# Prevalence and determinants of social frailty among community-dwelling empty-nest older adults in China

**DOI:** 10.3389/fpubh.2026.1860833

**Published:** 2026-07-21

**Authors:** Xia Zou, MingXiang Zheng, YuQing He, Hengxu Wang

**Affiliations:** 1 Mass Organizations Work Department, Central Hospital of Dalian University of Technology, Dalian Municipal Central Hospital, Dalian, China; 2 Ultrasound Imaging Department, Clinical Research Centre for Reproduction and Genetics in Hunan Province, Reproductive & Genetic Hospital of CITIC-Xiangya, Changsha, China; 3 NHC Key Laboratory of Human Stem Cell and Reproductive Engineering, School of Basic Medical Sciences, Central South University, Changsha, China; 4 School of Nursing, Changsha Medical University, Changsha, China

**Keywords:** community, current situation, empty nesters, influencing factors, social frailty

## Abstract

**Objective:**

To investigate the prevalence of social frailty and identify associated factors among community-dwelling empty-nest elders in China.

**Methods:**

A multistage random sampling strategy was employed to recruit 739 empty-nest older adults residing in urban communities in Changsha, China, between March and October 2023. Data were collected via structured questionnaires, including a general information form, the Social Frailty Index Scale, the Lubben Social Network Scale-6, the Family APGAR Index, UCLA Loneliness Scale – Short Form, and Geriatric Depression Scale – 5-item version.

**Results:**

The mean social frailty score was 1.64 ± 1.61. Among the participants, 38.02% were categorized as prefrail, 36.54% as frail, and 25.44% as non-frail. Multivariate logistic regression analysis revealed several protective factors against social frailty, including being married and cohabiting, being married but living apart, being enrolled in urban employment or urban resident medical insurance, participating in recreational activities, and having stronger family support. Conversely, risk factors for social frailty included being aged 60–69 years, having a monthly income below ¥1,000 or between ¥1,000–2,999, having multiple chronic conditions, experiencing social isolation, displaying depressive symptoms, and reporting greater levels of loneliness.

**Conclusion:**

Social frailty is common among community-dwelling empty-nest older adults in China. The key influencing factors include age, marital and cohabitation status, income level, medical insurance type, recreational activity, chronic disease burden, social isolation, family support, depressive symptoms, and loneliness. These findings underscore the importance of targeted public health strategies aimed at reducing social frailty in this vulnerable population.

## Introduction

Data from the Seventh National Population Census shows that the population aged 60 and above in China totaled 264 million at the end of 2020, constituting 18.7% of the overall population (1). As population aging accelerates, the number of empty-nest elders—defined as older adults living alone or solely with a spouse after their children have left home—rose to 118 million in 2020 and are projected to encompass nearly 90% of the older adults population by 2030 (2, 3). Rooted in family life cycle theory (4), the emergence of the “empty nest” household structure has introduced a range of psychosocial challenges. Empty-nest older adults individuals are particularly susceptible to “empty nest syndrome,” which is characterized by emotional distress, depressive symptoms, and profound loneliness arising from reduced social interaction (5). The weakening of traditional family-based support systems and diminished social connectedness have further intensified the risk of social frailty, presenting a pressing public health concern.

Fried et al. (6) formally conceptualized frailty as an aging-related clinical syndrome at the 1987 U.S. Federal Conference on Aging, characterized by reduced physiological reserves and heightened vulnerability to stressors resulting from accumulated health deficits. Frailty is commonly categorized into three domains: physical frailty, cognitive frailty, and social frailty. While physical and cognitive frailty have been widely examined in gerontological research, social frailty remains comparatively underexplored, particularly among empty-nest older adults individuals. Current literature establishes a significant association between social frailty in older populations and increased vulnerability to multiple detrimental outcomes, including loss of functional ability, impaired cognition, depressive disorders, and elevated mortality (7). Epidemiological data estimate social frailty prevalence rates of 21–38.1% in this group (8, 9), with growing evidence supporting its potential role as both predictor and contributing factor to physical frailty onset (9). However, owing to its subtle presentation and lack of specific clinical indicators, social frailty is often overlooked in both clinical practice and community-based assessments.

Social frailty in empty-nest older adults individuals is a complex, multidimensional condition encompassing inadequate access to social resources, reduced participation in social activities, weakened interpersonal relationships, and declining self-management abilities. The absence of co-residing children may lead to difficulties in managing daily life and maintaining independence, ultimately compromising quality of life (10). Additionally, a lack of emotional support and companionship exacerbates feelings of loneliness and social isolation (11). Financial insecurity is another contributing factor, particularly for those who previously relied on financial assistance from their children. On an individual level, social frailty is closely linked with increased risks of depression (12), anxiety (13), and diminished self-worth. Deteriorating physical health may further aggravate these conditions. On a broader societal scale, the growing burden of social frailty among the older adults poses significant challenges to healthcare systems and social welfare infrastructures, underscoring the need for responsive policy and service frameworks.

Given the increasing prevalence and multifactorial nature of social frailty among empty-nest older adults individuals, a more comprehensive understanding of its development, determinants, and broader implications is urgently needed. This study utilized a cross-sectional survey to examine the sense of social frailty and its correlates among empty-nesting older adults in the community. The findings can inform the development of targeted intervention strategies and supportive services that promote social integration, enhance quality of life, and support the creation of inclusive, age-friendly communities capable of addressing the unique needs of this growing population.

## Materials and methods

### Study participants

According to the 2020 edition of *medical statistics* edited by Sun Zhenqiu (People's Medical Publishing House), the sample size for multivariate analysis is typically 10–20 times the number of variables involved (14). This study included 27 influencing factors (20 items from the general information questionnaire, 2 dimensions from the Social Frailty Index, 2 dimensions from the Social Isolation Scale, and one dimension each from the Depression Scale, Family APGAR Index, and Loneliness Scale). Considering a 20% invalid response rate, the maximum required sample size was calculated as follows: *N* = (27 × 20) × (1 + 20%) = 648. To ensure sufficient statistical power, we aimed to collect as many responses as possible and ultimately obtained 739 completed questionnaires.

### Inclusion and exclusion criteria

From March and October 2023, a random sampling method was adopted in Hunan Province. Two administrative districts were randomly selected from six districts in Changsha city, and two streets were randomly selected from each district. Older adults individuals living alone or with a spouse who met the inclusion and exclusion criteria were selected as the study population.

The inclusion criteria were as follows: ① aged ≥ 60 years; ② defined as “empty-nest older adults,” i.e., living alone or with a spouse for more than six months; ③clear consciousness and ability to communicate; and ④ voluntary participation and ability to complete the survey.

The exclusion criteria were as follows: ① the presence of major diseases or terminal illness; ② a diagnosis of mental disorders; and ③ significant hearing, vision, or speech impairments affecting communication.

This study was approved by the Ethics Committee of Hunan Normal University (Approval No. 2023282), and informed consent was obtained from all participants.

### General information questionnaire

These data included the following: (1) Sociodemographic characteristics: age, sex, occupation, education level, personal monthly income, marital status, living arrangement, and medical insurance; (2) lifestyle and behavior: physical exercise and leisure activities; and (3) health-related data: chronic pain, history of falls, self-rated health, number of chronic diseases, and number of medications used.

### Social frailty index (SFI)

The SFI, developedby Teo et al. (6), is an index-based self-reported scale that assesses aspects of social activity, behavior, and resources. The Chinese version, adapted by Zhang Lulu et al. (15), contains 7 items in 2 dimensions: social participation and socioeconomic deprivation. Responses are scored dichotomously, with affirmative answers receiving 1 point and negative responses 0 points, yielding a total possible score between 0 and 7. Scores are interpreted as follows: 0 = no social frailty, 1 = prefrailty, and 2–7 = social frailty. Psychometric analysis demonstrated excellent reliability (α = 0.820, test-retest *r* = 0.958).

### Lubben social network scale-6 (LSNS-6)

Originally created by Lubben and colleagues (16), the LSNS-6 has become a frequently employed instrument for evaluating social isolation in older adults populations. Its Mandarin adaptation, which underwent validation by Chang's research team (17), comprises six questions categorized into two subscales: familial relationships (items 1–3) and social friendships (items 4–6). Subscale scores range 0–15 each, producing a composite score of 0–30. Clinical thresholds identify isolation risk at total scores < 12 or subscale scores < 6. The measure demonstrates excellent internal consistency (α = 0.921).

### Family APGAR index

Developed by Smilkstein (18), this scale consists of five items assessing adaptability, partnership, growth, affection, and resolve. Each item is rated on a 3-point Likert scale: “hardly ever” (0), “some of the time” (1), and “almost always” (2). Higher scores indicate better family support. Total scores of 7–10 indicate good family function, scores of 4–6 suggest moderate dysfunction, and scores of 0–3 reflect severe dysfunction (19). The Cronbach's α coefficient is 0.840.

### UCLA loneliness scale – short form (ULS-8)

This scale was developed by Hays et al. (20) based on the USLA-20 scale. The Loneliness Scale consists of 8 items, including 2 reverse-scored items and 6 positively scored items. Each item is rated on a scale from 1 to 4 (1 = Never, 2 = Rarely, 3 = Sometimes, 4 = Always). The total score ranges from 8 to 32, with higher scores indicating a greater degree of loneliness. The Cronbach's α coefficient of this scale is 0.831.

### Geriatric depression scale – 5-item version (GDS-5)

The Geriatric Depression Scale-5 (GDS-5), derived from the original 15-item version (21), comprises five screening questions. Items are scored dichotomously (1 = affirmative, 0 = negative), with item 1 requiring reverse scoring. Total scores (range: 0–5) demonstrate a positive correlation with depression severity, where scores ≥2 indicate probable depressive disorder. Psychometric evaluation confirms acceptable internal consistency (α = 0.80).

### Data collection

The study was conducted in community-based locations throughout Hunan Province. Research assistants, who received standardized training, administered the surveys using consistent verbal instructions to explain the study objectives and methodology. Written informed consent was acquired before questionnaire administration. All instruments were completed on-site with immediate quality checks, and participants were asked to clarify any omitted responses. From 750 distributed surveys, 739 valid questionnaires were returned, yielding a 98.4% valid response rate.

### Statistical analysis

The data management process involved dual independent data entry by researchers using Excel, followed by statistical analysis with SPSS 26.0. Continuous variables with normal distribution were expressed as mean ± SD and compared using parametric tests (*t*-tests/ANOVA), while non-parametric data were presented as median (min, max) and analyzed using Mann-Whitney *U* or Kruskal-Wallis tests. Categorical variables were summarized as counts (percentages). Social frailty variations across demographic and health-related categories were examined through non-parametric tests. Bivariate correlations were assessed using Pearson's method, while significant predictors of social frailty in community-based empty-nest older adults were identified through multivariate logistic regression modeling, with statistical significance set at α = 0.05.

## Results

### Demographic characteristics of community-dwelling empty-nest older adults individuals

The mean age of the community-dwelling empty-nest older adults individuals was 72.68 ± 6.76 years, with 17.59% aged 80 years or older. The most common body mass index (BMI) category was 18.5–23.9 kg/m^2^, accounting for 52.91% of the participants. In terms of gender distribution, 46.14% were male, and 53.86% were female. The majority of participants were married and cohabiting with their spouse (78.89%). With respect to educational attainment, the largest proportion had completed junior high school (41.27%).

A detailed distribution of social frailty scores across various demographic subgroups is presented in Table 1.

**Table 1:** Comparison of social frailty scores among community-dwelling empty-nest older adults with different demographic characteristics.

Variable	Total (*n*, %)	Non-frail (*n*, %)	Pre-frail (*n*, %)	Frail (*n*, %)	Frailty score (Mean ±SD)	Z/H value	*P* value
Age (years)						145.38	< 0.001
60–69	289 (39.11)	102 (35.29)	140 (48.44)	47 (16.26)	0.99 ± 1.12		
70–79	320 (43.30)	86 (26.88)	116 (36.25)	118 (36.88)	1.57 ± 1.54		
≥80	130 (17.59)	0 (0)	25 (19.23)	105 (80.77)	3.25 ± 1.61		
BMI (kg/m^2^)						3.255	0.196
< 18.5	22 (2.98)	3 (13.64)	6 (27.27)	13 (59.09)	2.32 ± 1.76		
18.5–23.9	391 (52.91)	104 (26.20)	143 (36.57)	144 (36.83)	1.60 ± 1.60		
24.0–27.9	257 (34.78)	71 (27.63)	97 (37.74)	89 (34.63)	1.62 ± 1.64		
≥28.0	6 (9.34)	10 (14.49)	35 (50.72)	24 (34.78)	1.71 ± 1.49		
Gender						−2.95	0.003
Male	341 (46.14)	92 (26.98)	149 (43.70)	100 (29.33)	1.41 ± 1.41		
Female	398 (53.86)	96 (24.12)	132 (33.17)	170 (42.71)	1.83 ± 1.75		
Marital status						220.356	< 0.001
Unmarried	2 (0.27)	0 (0)	1 (50.00)	1 (50.00)	1.50 ± 0.71		
Married and living together	583 (78.89)	187 (32.08)	261 (44.77)	135 (23.16)	1.19 ± 1.26		
Married but separated	20 (2.71)	1 (5.00)	8 (40.00)	11(55.00)	2.50 ± 1.82		
Divorced	19 (2.57)	0 (0)	6 (31.58)	13 (68.42)	2.47 ± 1.50		
Widowed	115 (15.56)	0 (0)	5 (4.35)	110 (95.65)	3.63 ± 1.59		
Education level						51.826	< 0.001
Illiterate	15 (2.03)	2 (13.33)	1 (6.67)	12 (80.00)	3.47 ± 2.10		
Primary school	105 (14.21)	3 (2.86)	28 (26.67)	74 (70.48)	2.97 ± 1.89		
Junior high school	305 (41.27)	91 (29.84)	114 (37.38)	100 (32.79)	1.46 ± 1.50		
High school	216 (29.23)	60 (27.78)	96 (44.44)	60 (27.78)	1.32 ± 1.31		
College or above	96 (12.99)	31 (32.29)	41 (42.71)	24 (25.00)	1.19 ± 1.24		
Graduate degree	2 (0.27)	1 (50.00)	1 (50.00)	0 (0)	0.50 ± 0.71		
Monthly income (RMB)						79.857	< 0.001
< 1,000	90 (12.18)	10 (11.11)	16 (17.78)	64 (71.11)	2.93 ± 1.97		
1,000–2,999	306 (41.41)	54 (17.65)	126 (41.18)	126 (41.18)	1.81 ± 1.58		
3,000–4,999	276 (37.35)	94 (34.06)	112 (40.58)	70 (25.36)	1.21 ± 1.32		
≥5,000	67 (9.07)	30 (44.78)	27 (40.30)	10 (14.93)	0.87 ± 1.13		
Medical insurance						11.119	0.004
Urban employee insurance	566 (76.59)	156 (27.56)	220 (38.87)	190 (33.57)	1.51 ± 1.53		
Urban resident insurance	95 (12.86)	31 (32.63)	56 (58.95)	71 (74.74)	2.04 ± 1.82		
Self-pay	2 (0.270)	0 (0)	0 (0)	2 (100.00)	3.33 ± 0.58		
Other	13(1.76)	1 (7.69)	5 (38.46)	7 (53.85)	2.00 ± 1.54		
Occupation						2.024	0.364
Worker	380 (51.42)	91 (23.95)	151 (39.74)	138 (36.32)	1.59 ± 1.56		
Farmer	55 (7.44)	8 (14.55)	20 (36.36)	27 (49.09)	2.13 ± 1.75		
Self–employed people	68 (9.02)	17 (25.00)	23 (33.82)	28 (41.18)	1.84 ± 1.75		
Civil servant	164 (22.19)	54 (32.93)	59 (35.98)	51 (31.10)	1.46 ± 1.60		
Other	72 (9.74)	18 (25.00)	28 (38.89)	26 (36.11)	1.71 ± 1.61		
Polypharmacy							
Yes	190 (25.71)	1 (0.53)	62 (32.63)	127 (66.84)	2.79 ± 1.58		
No	549 (74.29)	187 (34.06)	219 (39.89)	143 (26.05)	1.23 ± 1.42		
Multimorbidity						−13.4	< 0.001
Yes	321 (43.44)	0 (0)	142 (44.24)	179 (55.76)	2.54 ± 1.62		
No	418 (56.56)	188 (44.98)	139 (33.25)	91 (21.77)	0.94 ± 1.21		
Chronic pain						−12.217	< 0.001
Yes	350 (47.36)	31 (8.86)	120 (34.29)	199 (56.86)	2.32 ± 1.65		
No	389 (52.64)	157 (40.36)	161 (41.39)	71 (18.25)	1.02 ± 1.30		
Fall history						−7.263	< 0.001
Yes	77 (10.42)	2 (2.60)	18 (23.38)	57 (74.03)	2.78 ± 1.52		
No	662 (89.58)	186 (28.10)	263 (39.73)	213 (32.18)	1.50 ± 1.57		
Leisure activity						−15.997	< 0.001
Yes	434 (58.73)	188 (43.32)	177 (40.78)	69 (15.90)	0.82 ± 1.01		
No	304 (41.14)	0 (0)	104 (34.21)	201 (66.12)	2.79 ± 1.60		
Physical exercise						37.423	< 0.001
Never	83 (11.23)	12 (14.46)	26 (31.33)	45 (54.22)	2.27 ± 1.75		
Occasionally	254 (34.37)	52 (20.47)	86 (33.86)	116 (45.67)	1.91 ± 1.70		
Often	402 (54.40)	124 (30.85)	169 (42.04)	109 (27.11)	1.33 ± 1.45		
Group activities						248.388	< 0.001
Never participate	323 (43.71)	14 (4.33)	112 (34.67)	197 (60.99)	2.60 ± 1.67		
Occasionally participate	246 (33.29)	66 (26.83)	118 (47.97)	62 (25.20)	1.16 ± 1.13		
Often participate	141 (19.08)	87 (61.70)	45 (31.91)	9 (6.38)	0.51 ± 0.88		
Actively participate	29 (3.92)	21 (72.41)	6 (20.69)	2 (6.90)	0.45 ± 0.95		
Smoking						2.866	0.239
No	555 (75.10)	140 (25.23)	204 (36.76)	211 (38.02)	1.69 ± 1.65		
Quit smoking	45 (6.09)	4 (8.89)	21 (46.67)	20 (44.44)	1.98 ± 1.55		
Occasionally	37 (5.01)	7 (18.92)	17 (45.95)	13 (35.14)	1.68 ± 1.62		
Frequently	102 (13.80)	37 (36.27)	39 (38.24)	26 (25.49)	1.18 ± 1.32		
Alcohol use						2.177	0.337
No	622 (84.17)	159 (25.56)	230 (36.98)	233 (37.46)	1.33 ± 1.42		
Quit drinking	19 (2.57)	2 (10.53)	7 (36.84)	10 (52.63)	1.58 ± 1.02		
Occasionally	64 (8.66)	18 (28.13)	27 (42.19)	19 (29.69)	1.00 ± 0.99		
Frequently	34 (4.60)	9 (26.47)	17 (50.00)	8 (23.53)	0.79 ± 1.10		
Night sleep duration						165.664	< 0.001
< 5 h	248 (33.56)	12 (4.84)	69 (27.82)	167 (67.34)	2.70 ± 1.64		
5–8 h	465 (62.92)	163 (35.05)	203 (43.66)	99 (21.29)	1.12 ± 1.31		
>8 h	26 (3.52)	13 (50.00)	9 (34.62)	4 (15.38)	0.73 ± 0.96		
Self–rated health						41.555	< 0.001
Satisfied	114 (15.43)	45 (39.47)	38 (33.33)	31 (27.19)	1.18 ± 1.35		
Basically satisfied	526 (71.18)	138 (26.24)	212 (40.30)	176 (33.46)	1.52 ± 1.52		
Average	67 (9.07)	4 (5.97)	19 (28.36)	44 (65.67)	2.84 ± 1.98		
Not very satisfied	24 (3.25)	0 (0)	7 (29.17)	17 (70.83)	2.92 ± 1.50		
Dissatisfied	8 (1.08)	1 (12.50)	5 (62.50)	2 (25.00)	1.63 ± 1.60		

### Relationships among social frailty, social isolation, family care, depression, and loneliness among community-dwelling empty-nest older adults individuals

The mean social frailty score among the participants was 1.64 ± 1.61. Among the total sample, 188 individuals were classified as non-frail, whereas 38.02% and 36.54% were identified as prefrail and frail, respectively. The mean scores for social isolation, family functioning, depression, and loneliness were 14.88 ± 5.75, 7.20 ± 2.64, 1.33 ± 1.50, and 15.71 ± 5.94, respectively.

Pearson correlation analysis revealed that social frailty was significantly correlated with social isolation (*r* = −0.75, *P* < 0.01), family function (*r* = −0.75, *P* < 0.01), loneliness (*r* = 0.77, *P* < 0.01), and depression (*r* = 0.76, *P* < 0.01), as detailed in Table 2.

**Table 2:** Correlation analysis of social frailty among community-dwelling empty-nest older adults.

Variable	Social isolation	Family care	Loneliness	Depression
Social frailty	−0.75^**^	−0.75^**^	0.77^**^	0.76^**^
Social life	−0.72^**^	−0.72^**^	0.73^**^	0.74^**^
Socioeconomic deprivation	−0.54^**^	−0.56^**^	0.57^**^	0.54^**^

^**^P < 0.01.

### Multivariate logistic regression analysis of social frailty among community-dwelling empty-nest older adults individuals

To further explore the determinants of social frailty, an ordinal regression analysis was conducted. Social frailty status was treated as the dependent variable and categorized as 0 = non-frail, 1 = prefrail, and 2 = frail. Independent variables included factors found to be statistically significant in the univariate analysis (*P* < 0.05), as well as psychosocial variables such as social isolation, family function, depression, and loneliness. The proportional odds assumption was tested using the parallel lines test, which yielded a non-significant result (*P* > 0.05), indicating that the assumption holds. The coding scheme for all the variables is provided in Table 3.

**Table 3:** Coding of independent variables for multivariate logistic regression analysis (*n* = 739).

Code	Variable	Coding method
1	Age	0 = ≥80; 1 = 70–79; 2 = 60–69
2	Gender	0 = Female; 1 = Male
3	Marital status	0 = Widowed; 1 = Divorced; 2 = Married but separated; 3 = Married and living together; 4 = Unmarried
4	Education level	0 = Graduate degree; 1 = College or above; 2 = High school; 3 = Junior high school; 4 = Primary school; 5 = Illiterate
5	Monthly income	0 = ≥5000; 1 = 3000−4999; 2 = 1000−2999; 3 = < 1000
6	Medical insurance type	Urban employee insurance = (1,0,0); Urban resident insurance = (0,1,0); Self-pay = (0,0,1); Other = (0,0,0)
7	Polypharmacy	0 = No; 1 = Yes
8	Chronic disease coexistence	0 = No; 1 = Yes
9	Chronic pain	0 = No; 1 = Yes
10	Falls	0 = No; 1 = Yes
11	Recreational activities	0 = No; 1 = Yes
12	Physical exercise	0 = Often; 1 = Occasionally; 2 = Never
13	Group activities	0 = Actively participate; 1 = Often participate; 2 = Occasionally participate; 3 = Never participate
14	Nighttime sleep duration	0 = >8 h; 1 = 5–8 h; 2 = < 5 h
15	Self-rated health status	0 = Dissatisfied; 1 = Not very satisfied; 2 = Average; 3 = Basically satisfied; 4 = Satisfied
16	Social isolation	Original values
17	Family care	Original values
18	Depression	Original values
19	Loneliness	Original values
20	Social frailty	0 = No social frailty; 1 = Pre-social frailty; 2 = Social frailty

The results revealed several protective factors against social frailty, including being married and living with a spouse, being married but living apart, being enrolled in urban employee or urban resident medical insurance, participating in recreational activities, and having higher levels of family support. Conversely, risk factors for social frailty included being aged 60–69 years, having a monthly income below ¥1,000 or between ¥1,000–2,999, having multiple chronic diseases, having higher levels of social isolation, having depressive symptoms, and having loneliness. A detailed summary of the regression results is provided in Table 4.

**Table 4:** Logistic regression analysis of factors influencing social frailty among community-dwelling empty-nest older adults.

Variable	β	Std. Error	Wald χ^2^	P	OR	95% CI
Age (years)						
60–69	0.701	0.337	4.317	0.038	2.016	1.041–4.067
70–79	0.525	0.323	2.651	0.104	1.69	0.899–3.251
≥80 (Ref.)	1					
Gender						
Male	0.02	0.14	0.019	0.889	1.02	0.775–1.342
Female (Ref.)	1					
Marital status						
Unmarried	−1.722	1.161	2.199	0.138	0.179	0.018–1.740
Married but separated	−1.643	0.668	6.055	0.014	0.193	0.052–0.716
Married and living together	−2.584	0.517	24.946	< 0.001	0.075	0.027–0.208
Divorced	−1.251	0.661	3.585	0.058	0.286	0.078–1.045
Widowed (Ref.)	1					
Education level						
Illiterate	1.794	1.514	1.404	0.236	6.013	0.309–116.863
Primary school	1.988	1.433	1.923	0.166	7.301	0.440–121.146
Junior high school	0.524	1.41	0.138	0.71	1.689	0.106–26.816
High school	0.501	1.411	0.126	0.722	1.65	0.104–26.206
College or above	0.412	1.415	0.085	0.771	1.51	0.094–24.167
Graduate degree (Ref.)	1					
Monthly income (RMB)						
< 1,000	1.133	0.388	8.531	0.003	3.105	1.452–6.639
1,000–2,999	0.601	0.258	5.416	0.02	1.824	1.100–3.028
3,000–4,999	0.433	0.236	3.355	0.067	1.542	0.970–2.452
≥5,000 (Ref.)	1					
Medical insurance						
Urban employee insurance	−1.665	0.566	8.637	0.003	0.189	0.062–0.574
Urban resident insurance	−2.215	0.591	14.03	< 0.001	0.109	0.034–0.348
Self-pay	0.689	7.481	0.008	0.927	1.992	0–4643597.746
Other (Ref.)	1					
Polypharmacy						
Yes	0.189	0.251	0.567	0.452	1.208	0.739–1.976
No (Ref.)	1					
Chronic disease coexistence						
Yes	2.174	0.221	96.773	< 0.001	8.793	5.703–13.558
No (Ref.)	1					
Chronic pain						
Yes	−0.133	0.17	0.615	0.433	0.875	0.627–1.221
No (Ref.)	1					
Falls						
Yes	0.231	0.3	0.59	0.442	1.26	0.699–2.268
No (Ref.)	1					
Recreational activities						
Yes	−1.232	0.228	29.207	< 0.001	0.295	0.189–0.461
No (Ref.)	1					
Physical exercise						
Never	0.277	0.25	1.225	0.268	1.319	0.808–2.153
Occasionally	0.2	0.151	1.756	0.185	1.221	0.908–1.642
Often (Ref.)	1					
Group activities						
Never participate	0.004	0.385	0	0.993	1.004	0.472–2.134
Occasionally participate	0.378	0.347	1.187	0.276	1.459	0.739–2.883
Often participate	0.016	0.336	0.002	0.962	1.016	0.526–1.964
Actively participate (Ref.)	1					
Night sleep (hours)						
< 5 h	−0.471	0.384	1.506	0.22	0.624	0.294–1.324
5–8 h	−0.273	0.336	0.658	0.417	0.761	0.394–1.471
>8 h (Ref.)	1					
Self–rated health						
Satisfied	0.826	0.669	1.528	0.216	2.284	0.616–8.474
Basically satisfied	0.795	0.653	1.484	0.223	2.214	0.627–7.819
Average	1.108	0.708	2.453	0.117	3.028	0.757–12.122
Not very satisfied	0.023	0.806	0.001	0.977	1.023	0.211–4.973
Dissatisfied (Ref.)	1					
Social isolation	−0.091	0.026	12.303	< 0.001	2	1.548–2.585
Family care level	−0.18	0.059	9.138	0.003	0.835	0.742–0.939
Depression	0.22	0.105	4.367	0.037	1.245	1.014–1.528
Loneliness	0.138	0.028	24.655	< 0.001	1.148	1.088~1.213

## Discussion

The findings of this study revealed that the average social frailty score among community-dwelling empty-nest older adults individuals was 1.64 ± 1.61, with the majority classified as either prefrail (38.02%) or socially frail (36.54%). These results are consistent with those reported by Yang Qingjian (22) and Ko et al. (23). In contrast, Chen Yingwei et al. (24) reported a lower prevalence of 25.91% among the general community-dwelling older adults population, whereas Zhao Ou (25) reported a rate of only 12.0% in hospitalized older adults. These comparisons underscore the disproportionately higher prevalence of social frailty among empty-nest elders, which is likely attributable to the absence of coresiding children. Many in this population live alone or solely with a spouse, which limits opportunities for social interaction and increases vulnerability to negative emotional states such as loneliness and isolation—key contributors to social frailty (22).

The ordinal regression analysis revealed that older adults aged 60–69 years had higher odds of social frailty compared with those aged ≥80 years. This suggests that younger older adults (60–69 years) are at greater risk of social frailty than the oldest-old group (≥80 years). In contrast to the findings by Makizako et al., our results show a different pattern (26), One possible explanation is that the oldest-old individuals in our sample may have developed coping strategies or received more social support due to their advanced age, whereas the relatively younger older adults (60–69 years) might still face significant life transitions (e.g., retirement, children leaving home) that increase their vulnerability to social frailty. In addition, our results support those of Andraw et al. (3), showing that being married—whether cohabiting or living separately—serves as a protective factor. The emotional support and companionship provided by a spouse can enhance psychological wellbeing and promote healthier aging.

Access to healthcare resources also influences social frailty outcomes. The possession of urban employee or urban resident medical insurance was found to be protective, which is consistent with findings by St et al. (27). For older adults with limited financial resources, high healthcare costs may restrict access to essential services, exacerbating psychological distress and perceived helplessness and thereby increasing the risk of social frailty.

Economic status was another critical factor. Participants with monthly incomes below ¥1,000 or between ¥1,000–2,999 presented significantly higher odds of social frailty, corroborating prior research by Ma et al. (28). Limited financial resources can constrain access to adequate nutrition, housing, medical care, and social participation, all of which contribute to increased vulnerability.

The presence of multiple chronic conditions has emerged as one of the most robust risk factors. Compared with those without chronic diseases, empty-nest elders with comorbidities had an 8.793-fold greater risk of social frailty, which is consistent with findings by Zhang Zhiyue et al. (29). Age-related physiological decline and reduced immune function often necessitate polypharmacy, which may result in adverse drug reactions, further compromising physical resilience and accelerating the progression of social frailty (30).

Participation in srecreational activities served as a protective factor against social frailty. Engagement in leisure and social activities provides opportunities for interpersonal interaction and community integration, contributing to improved psychological wellbeing and reduced social isolation (31).

Consistent with prior studies by Maltby et al. (32), social isolation was significantly associated with social frailty. As older adults retire and lose family caregiving roles, their social networks may shrink, leading to diminished social identity and function. Reduced social engagement has been recognized as a precursor to physical frailty and functional disability (33).

Family support—particularly emotional and financial support—was shown to buffer against social frailty, which is consistent with findings by Prabhu et al. (34). Strong family connections enhance psychological security and provide practical assistance, thereby mitigating the adverse effects of social detachment and dependence.

Depressive symptoms were strongly associated with social frailty. Older adults with more severe depressive symptoms were significantly more likely to be socially frail. International evidence suggests that depression increases the risk of social frailty by up to 16.7 times (35), and among the various domains of frailty, social frailty is both a predictor and a consequence of depressive states (36, 37).

Furthermore, loneliness was positively correlated with the severity of social frailty. Even in cases where financial support from children is maintained, persistent emotional loneliness and inadequate social connectedness can significantly impair mental health and exacerbate frailty (38).

Collectively, these findings highlight the multifactorial nature of social frailty among empty-nest older adults individuals. Addressing this issue requires coordinated efforts at both the societal and family levels. Interventions should focus on strengthening community-based support systems, enhancing opportunities for social participation, promoting family involvement, and improving access to psychological and mental health services. Such measures are essential for fostering resilience, enhancing life satisfaction, and ultimately improving overall quality of life in this vulnerable population.

## Limitations

This study has several limitations. First, the data were collected from only four communities in Changsha city, which limits the geographical generalizability of the findings. Additionally, the uneven distribution of participants across subgroups may reduce the representativeness of the sample, making it difficult to generalize the results to the broader population of older adults individuals in Hunan Province. Second, the study employed a cross-sectional design, which precludes the establishment of causal relationships and allows only the identification of associations between variables and social frailty. In particular, it remains unclear whether depression and loneliness lead to social frailty or whether social frailty triggers these negative psychological states; bidirectional or reverse causal pathways are possible. Therefore, stronger emphasis is placed on the need for longitudinal studies to clarify the temporal sequence and causal directions among these variables. Furthermore, expanding the scope of future research to include a larger and more diverse sample is recommended to increase the generalizability of the findings. Further exploration of this age-related impact is necessary to inform more targeted interventions and policy planning.

## Data Availability

The original contributions presented in the study are included in the article/supplementary material, further inquiries can be directed to the corresponding author.
